# Supplementary material for: Cigarette smoke aggravates asthma by inducing memory-like type 3 innate lymphoid cells
Source: Nat Commun. 2022 Jul 4;13:3852. doi: 10.1038/s41467-022-31491-1 (PMC9253141; doi:10.1038/s41467-022-31491-1)
Supplement: Supplementary file 2 — Supplementary Information [file 41467_2022_31491_MOESM2_ESM.pdf]

Cigarette smoke aggravates asthma by inducing memory-like type 3 innate lymphoid cells

Supplementary Figure 1

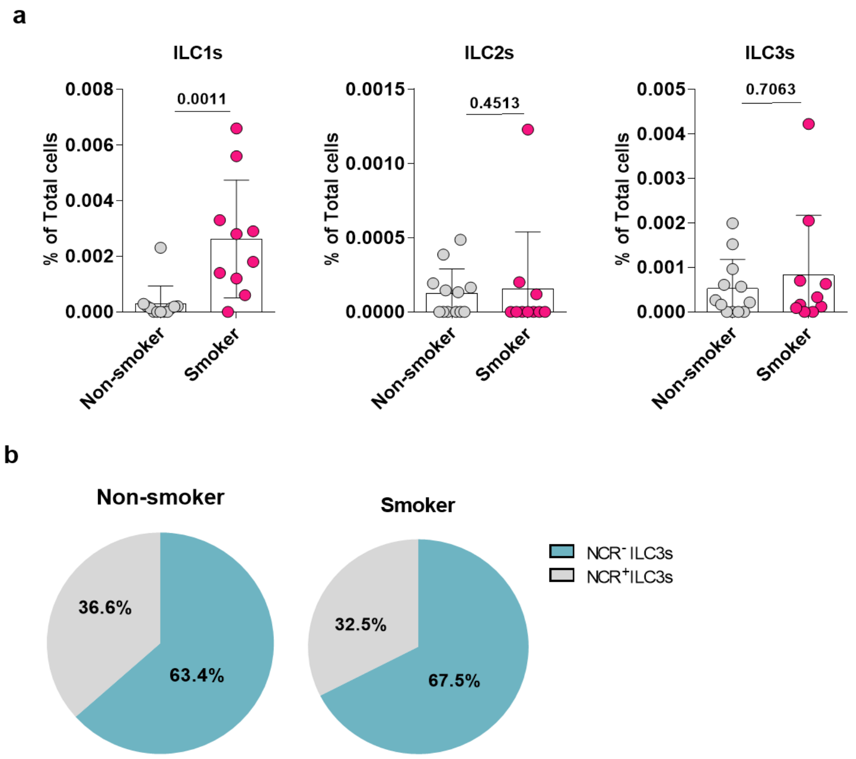

Supplementary Figure 1. ILC1s in induced sputum from healthy controls increase in smokers. **a**. Comparison of each subset of ILCs between non-smoking and smoking individuals in healthy controls. **b**. Proportion of ILC3s (ST-2$^-$ C-kit$^+$) in induced sputum of non-smoking and smoking healthy controls that are NCR$^-$ (NKp44$^-$) and NCR$^+$ (NKp44$^+$). Each dot represents individual subjects. Sample size of non-smokers, n = 12; smokers, n = 10 for supplementary figure 1a. The non-smokers and smokers in healthy controls were compared by two-tailed Mann-Whitney U test (supplementary figure 1a). The data are presented as mean ± standard deviation. $p < 0.05$ is considered as significant.

Supplementary Figure 2

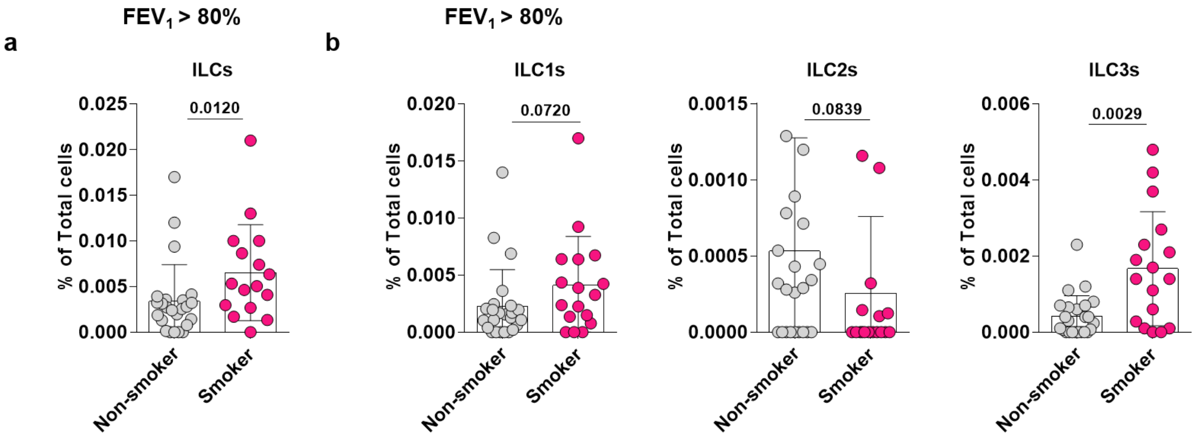

Supplementary Figure 2. Smokers with normal FEV_1 (%) have higher ILC3s in induced sputum than non-smokers with normal FEV_1 (%) from asthma patients. Asthmatics with normal FEV_1 (%) have more than 80% of FEV_1 (%). **a**. Comparison of frequency of total ILCs between non-smoking and smoking patients from asthmatics. **b**. Comparison of ILC1s, ILC2s, and ILC3s between non-smokers and smokers from asthmatics with normal FEV_1 (%). Each dot represents individual subjects. Sample size of non-smokers, n = 24; smokers, n = 16 for supplementary figure 2a, non-smokers, n = 24; smokers, n = 17 for ILC1s and ILC3s in supplementary figure 2b, non-smokers, n = 24; smokers, n = 18 for ILC2s in supplementary figure 2b. The non-smokers and smokers in asthma patients with normal FEV_1(%) were compared by two-tailed Mann-Whitney U test. The data are presented as mean ± standard deviation. $p < 0.05$ is considered as significant.

Supplementary Figure 3

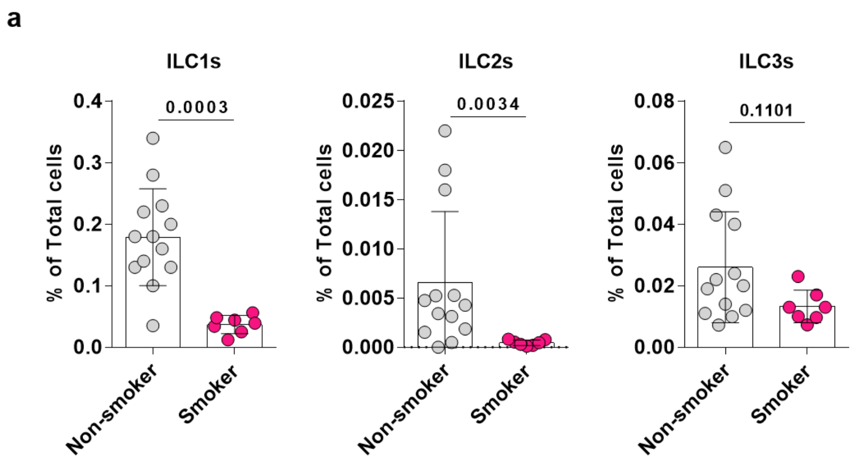

Supplementary Figure 3. Peripheral blood ILCs decreases in smokers among healthy controls. a. Comparison of each type of ILCs from healthy controls between non-smokers and smokers. Each dot represents individual subjects. Sample size of the non-smokers, n = 13; smokers, n = 7 for supplementary figure 3a. The non-smokers and smokers in healthy controls were compared by two-tailed Mann-Whitney U test. The data are presented as mean ± standard deviation. $p < 0.05$ is considered as significant.

Supplementary Figure 4

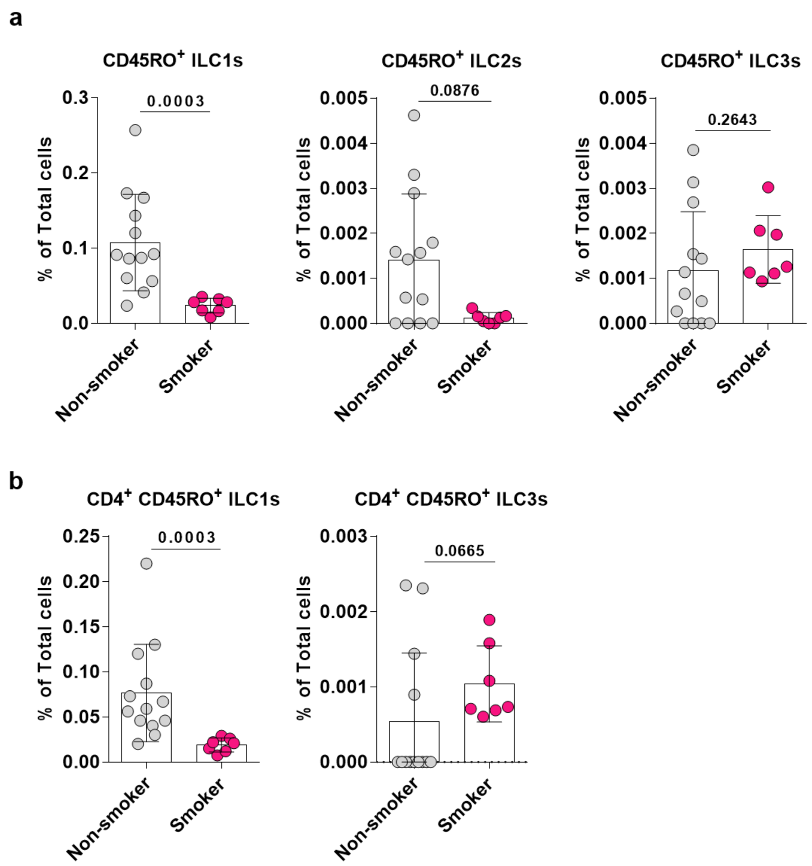

Supplementary Figure 4. **CD45RO and CD4 expressions from peripheral blood ILC3s were comparable in smokers among healthy controls. a**. Comparison of CD45RO-expressing ILCs between non-smoking and smoking individuals in healthy controls. **b**. Comparison of $CD4^+CD45RO^+$ILC1s or ILC3s between non-smokers and smokers in healthy controls. Each dot represents individual subjects. Sample size of non-smokers, n =13; smokers, n = 7 for $CD45RO^+$ILC1s and $CD45RO^+$ILC3s in supplementary figure 4a and $CD45RO^+CD4^+$ILC1s and $CD45RO^+CD4^+$ILC3s in supplementary figure 4b, non-smokers, n = 12; smokers, n = 7 for $CD45RO^+$ILC2s in supplementary figure 4a. The non-smokers and smokers in healthy controls were compared by two-tailed Mann-Whitney U test. The data are presented as mean ± standard deviation. $p < 0.05$ is considered as significant.

Supplementary Figure 5

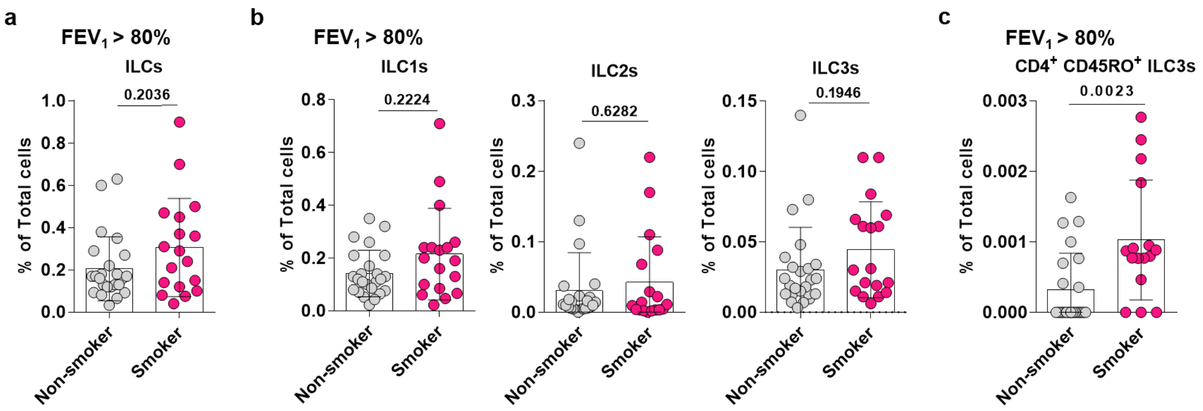

Supplementary Figure 5. CD4$^+$CD45RO$^+$ILC3s in blood are increased in smokers from asthmatics with normal FEV$_1$ (%). Asthmatics with normal FEV$_1$ (%) have more than 80% of FEV$_1$ (%). **a-b**. Comparison of frequency of circulating total ILCs (**a**), ILC1s, ILC2s, and ILC3s (**b**) between non-smokers and smokers from asthmatics with normal FEV$_1$ (%). **c**. Comparison of frequency of CD4$^+$CD45RO$^+$ILC3 subsets in blood between non-smokers and smokers in asthmatics with normal FEV$_1$ (%). Each dot represents individual subjects. Sample size of non-smokers, n = 24; smokers, n = 18 for supplementary figure 4a, b, non-smokers, n = 23; smokers, n = 16 for supplementary figure 4c. The non-smokers and smokers in asthma patients were compared by two-tailed Mann-Whitney U test. The data are presented as mean ± standard deviation. $p < 0.05$ is considered as significant.

Supplementary Figure 6

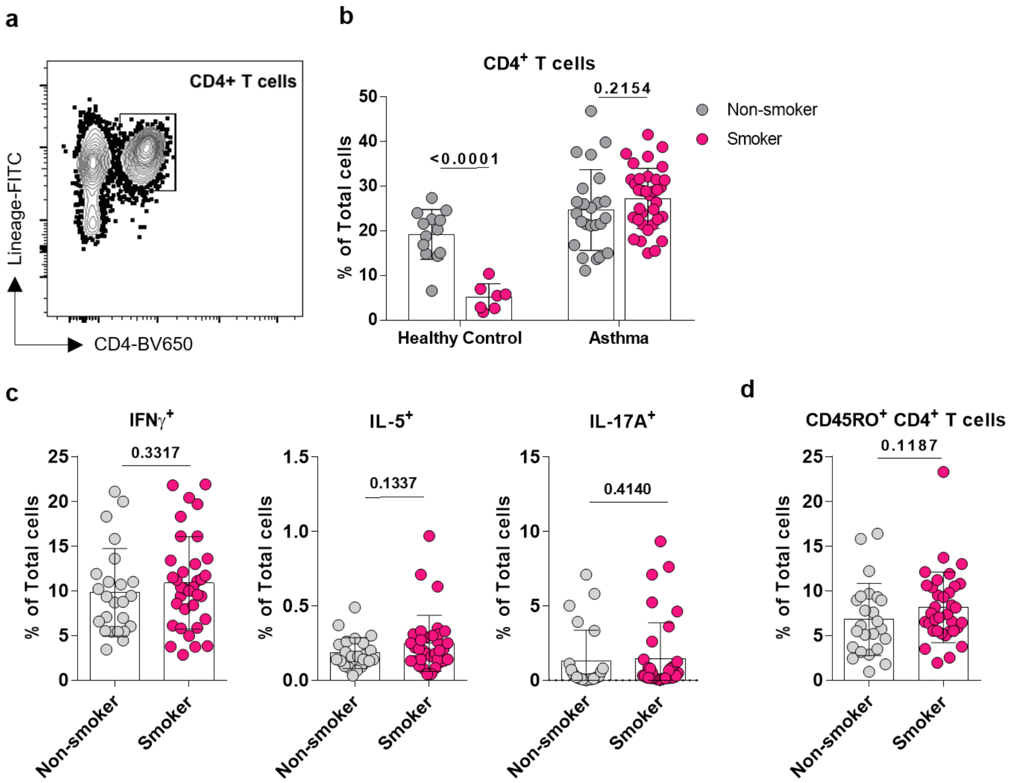

Supplementary Figure 6. Circulating CD4⁺ T cells have no differences between non-smokers and smokers from asthmatics. a. Gating strategy of CD4⁺ T cells from peripheral blood. **b**. Comparison of frequency of CD4⁺ T cells in blood between non-smoking and smoking patients with asthma; non-smoking and smoking individuals in healthy controls. **c**. Comparison of proportion of IFNγ, IL-5, and IL-17A⁺ T cells in blood between non-smokers and smokers in asthmatics. **d**. Comparison of frequency of CD45RO⁺CD4⁺ T cells in blood between non-smokers and smokers in asthmatics. Each dot represents individual subjects. Sample size of non-smoking healthy controls, n = 13; smoking healthy controls, n = 7; non-smoking asthma patients, n = 24; smoking asthma patients, n = 35 for supplementary figure 6b, non-smokers, n = 24; smokers, n = 35 for supplementary figure 6c, d. The non-smokers and smokers in the asthma patients or healthy individuals were compared by multiple t-tests (supplementary figure 6b). The non-smokers and smokers in asthma patients were compared by two-tailed Mann-Whitney U test (supplementary figure 6c, d). The data are presented as mean ± standard deviation. $p < 0.05$ is considered as significant.

Supplementary Figure 7

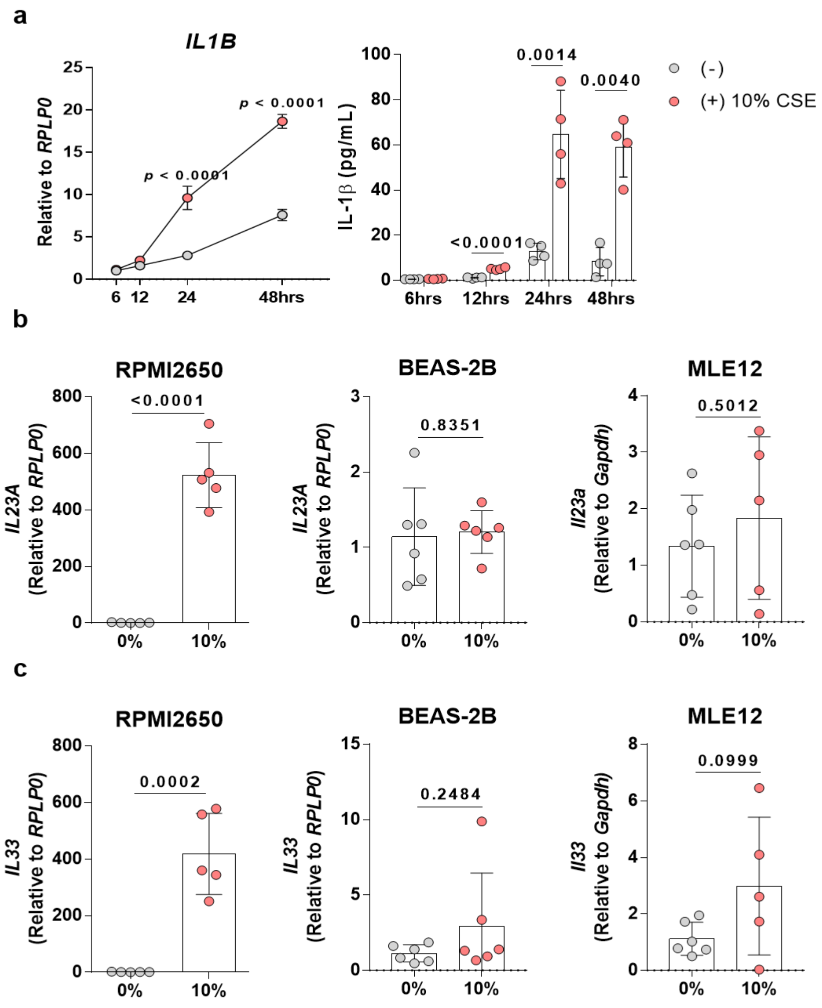

Supplementary Figure 7. Smoking induced stimulatory cytokine expressions. a. Comparison of CSE-treated and untreated A549 cells *IL1B* genes and protein expressions. The CSE concentrations ranged from 0% to 10% and cells were cultured for 6 to 48 hours. **b-c**. Comparison of *IL23A* (**b**), *IL33* (**c**) expression from CSE-treated and untreated RPMI2650, BEAS-2B, and MLE12 cell lines. Sample size of each time points with or without 10% CSE treatment, n = 4 respectively for supplementary figure 7a, 0% CSE treated, n = 5; 10% CSE treated, n = 5 in RPMI2650, 0% CSE treated, n = 6; 10% CSE treated, n = 6 in BEAS-2B, and 0% CSE treated, n = 6; 10% CSE treated, n = 5 in MEL12 for supplementary figure 7b, c. Each of the data was the representative data from more than twice replications. The CSE-treated A549 cells were compared to the untreated cells at the same time-point by multiple t test (supplementary figure 7a). *IL23A* and *IL33* expressions between CSE-treated and untreated cell lines were compared by two-tailed unpaired t test (supplementary figure 7b, c). The data are presented as mean ± standard deviation. $p < 0.05$ is considered as significant.

Supplementary Figure 8

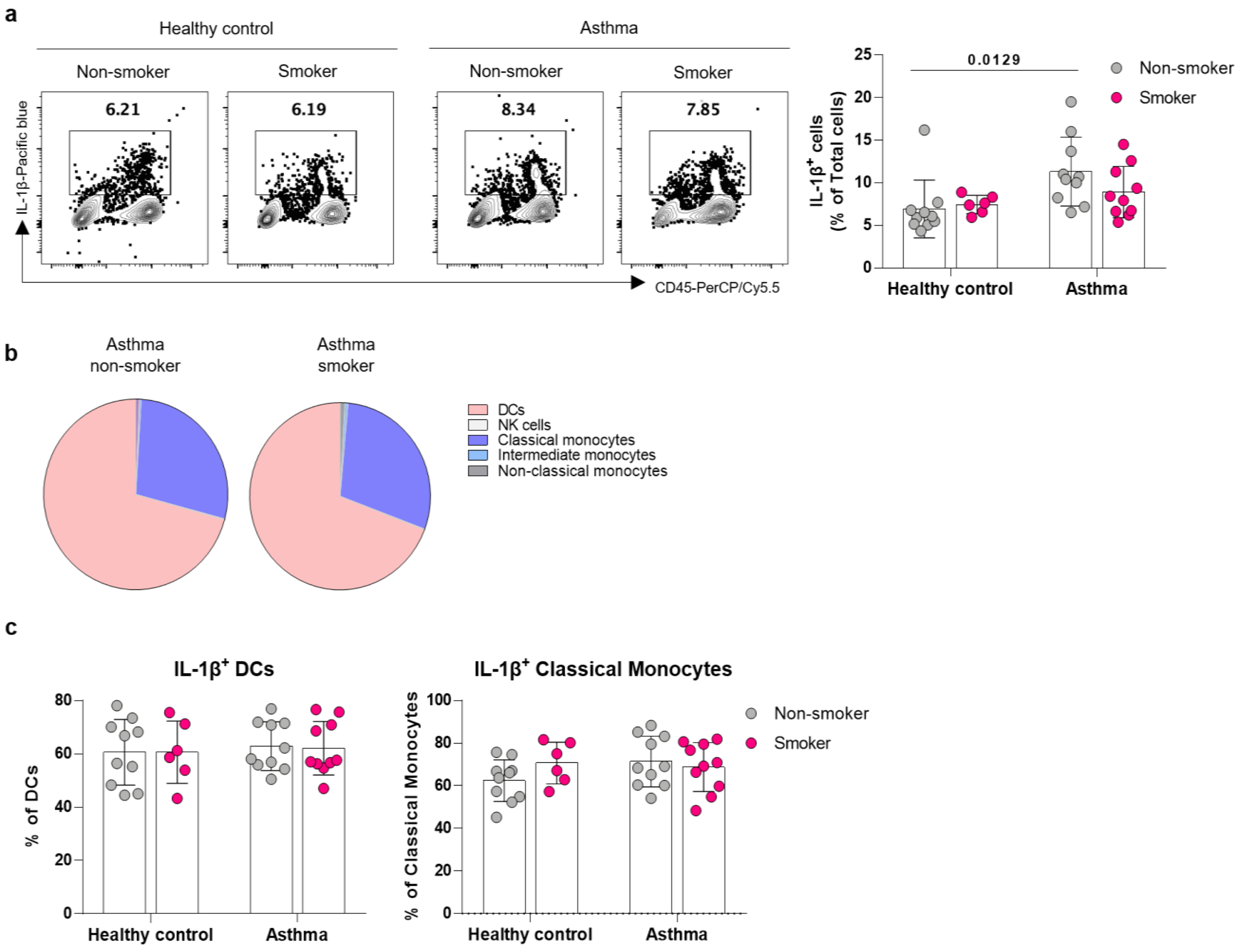

Supplementary Figure 8. Effect of smoking in IL-1β production from PBMCs. a. Comparison of IL-1β^{+} cells between non-smokers and smokers from healthy controls or asthma patients. **b**. Composition of IL-1β^{+} immune cells from PBMCs of asthmatic non-smokers and asthmatic smokers. Each cell was primary gated as IL-1β^{+} cells and further divided as described. Dendritic cells (DC): CD45^{+}CD11c^{+}HLA-DR^{+} cells; natural killer (NK) cells: CD45^{+}CD11c^{-}HLA-DR^{-}CD56^{+} cells; classical monocytes: CD45^{+}CD11c^{-}HLA-DR^{+}CD56^{-}CD14^{+}CD16^{-} cells; intermediate monocytes: CD45^{+}CD11c^{-}HLA-DR^{+}CD56^{-}CD14^{+}CD16^{+} cells; and non-classical monocytes: CD45^{+}CD11c^{-}HLA-DR^{+}CD56^{-}CD14^{-}CD16^{+} cells. **c.** Comparison of proportion of IL-1β^{+} cells from DCs or classical monocytes in between non-smokers and smokers from healthy controls or asthma patients. Each dot represents individual subjects. Sample size of non-smoking healthy controls, n = 10; smoking healthy controls, n = 6; non-smoking asthma patients, n = 10; smoking asthma patients, n = 10 for supplementary figure 8a, c. The smokers and non-smokers from healthy controls and asthma patients were compared by Two-way ANOVA (supplementary figure 8a, c). The data are presented as mean ± standard deviation. $p < 0.05$ is considered as significant.

Supplementary Figure 9

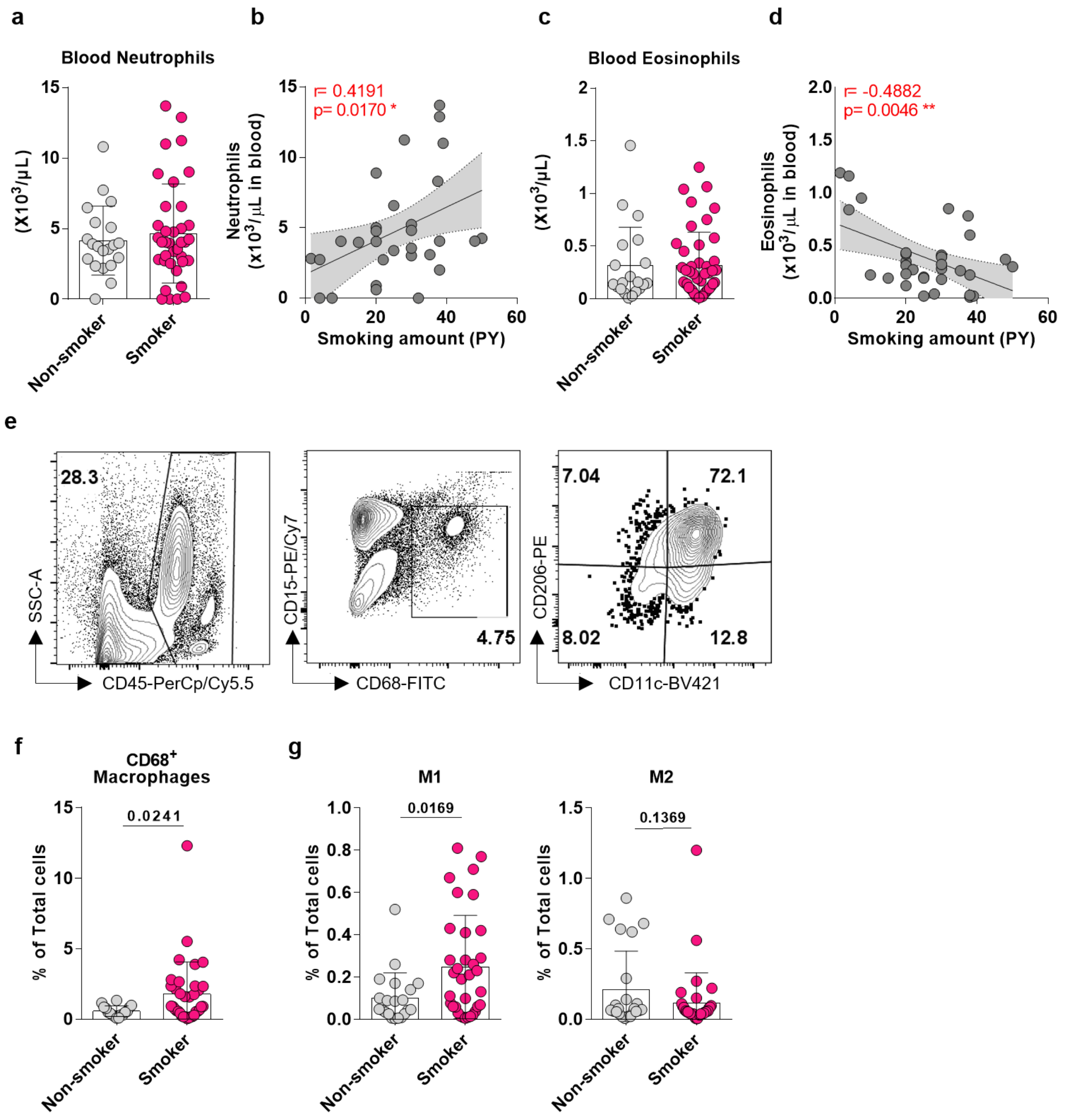

Supplementary Figure 9. M1 macrophages increase in induced sputum of smokers from asthma patients, not circulating neutrophils and eosinophils. a. Comparison of circulating neutrophils in peripheral blood between non-smoking and smoking individuals in asthma patients. **b**. Correlation between smoking amount (Pack-Year; PY) and circulating neutrophils count in blood. **c**. Comparison of circulating eosinophils in peripheral blood between non-smoking and smoking in asthma patients. **d**. Correlation between smoking amount (Pack-Year; PY) and circulating eosinophil count in blood. **e**. Gating strategy of macrophages in induced sputum. $CD45^{+}CD68^{+}$ cells are total macrophage. Among macrophages, $CD11c^{+}CD206^{-}$ cells are M1 macrophages and $CD11c^{-}CD206^{+}$ cells are M2 macrophages. **f**. Comparison of total macrophages in induced sputum between non-smokers and smokers in asthma patients. **g**. Comparison of frequency of M1 and M2 macrophages in induced sputum between non-smokers and smokers in asthmatics. Each dot represents individual subjects. Sample size of non-smokers, n = 20; smokers, n = 37 for supplementary figure 9a, non-smokers, n = 20; smokers, n = 44 for supplementary figure 9c, non-smokers, n = 16; smokers, n = 35 for supplementary figure 9f, non-smokers, n = 21; smokers, n = 34 for M1, and non-smokers, n = 23; smokers, n = 35 for M2 in supplementary figure 9g. The non-smokers and smokers in asthma patients were compared by two-tailed Mann-Whitney U test (supplementary figure 9a, c, f, g). Correlation analyses were conducted with Spearman r correlation test, dotted line represents 95% confidence interval (supplementary figure 9b, d). The data are presented as mean ± standard deviation. $p < 0.05$ is considered as significant.

Supplementary Figure 10

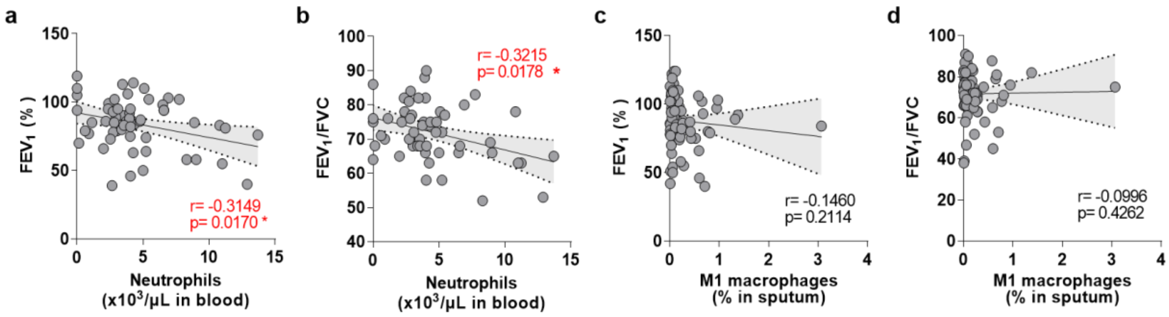

Supplementary Figure 10. Blood neutrophils and sputum M1 macrophage have less correlation with asthma severity. a-b. Correlation between the number of circulating neutrophils and clinical indices of asthma severity, FEV_1 (%) (**a**) and FEV_1/FVC (**b**). **c-d**. Correlation between the frequency of M1 macrophages in sputum and FEV_1 (%) (**c**) and FEV_1/FVC (**d**). Each dot represents individual subjects. Correlation analyses were conducted with Spearman r correlation test, dotted line represents 95% confidence interval. $p < 0.05$ is considered as significant.

Supplementary Table 1

Antibodies	Source	Identifier	Clone	Dilution factor
anti-CD45	BD Bioscience	Cat.: 564105	Clone: HI30	500:1
anti-CD3ε	BioLegend	Cat: 300406	Clone: UCHT1	300:1
anti-CD11c	BioLegend	Cat: 301604	Clone: 3.9	300:1
anti-CD11b	BioLegend	Cat: 301330	Clone: ICRF44	300:1
anti-CD14	BioLegend	Cat: 325604	Clone: HCD14	300:1
anti-CD19	BioLegend	Cat: 302206	Clone: HIB19	300:1
anti-CD49b	BioLegend	Cat: 359306	Clone: P1E6-C5	300:1
anti-FcεRIα	BioLegend	Cat: 334608	Clone: AER-37	300:1
anti-CD68	BioLegend	Cat: 333806	Clone: Y1/82A	200:1
anti-CD117	BioLegend	Cat: 313216	Clone: 104D2	200:1
anti-CD127	BioLegend	Cat: 351320	Clone: A019D5	200:1
anti-CD206	BioLegend	Cat: 321106	Clone: 15-2	200:1
anti-HLA-DR	BioLegend	Cat: 307610	Clone: L243	200:1
anti-CD4	BioLegend	Cat: 317436	Clone: OKT4	200:1
anti-CD45RO	BioLegend	Cat: 304245	Clone: UCHL1	200:1
anti-CD45RA	BioLegend	Cat: 304150	Clone: HI100	200:1
anti-CD56	BioLegend	Cat: 318305	Clone: HCD56	200:1
anti-CD16	BioLegend	Cat: 302012	Clone: 3G8	200:1
anti-NKp44	BioLegend	Cat: 325110	Clone: P44-8	200:1
anti-IFNγ	BioLegend	Cat: 502527	Clone: 4S.B3	100:1
anti-IL-5	BioLegend	Cat: 504311	Clone: TRFK5	100:1
anti-IL-17A	BioLegend	Cat: 512333	Clone: BL168	100:1
anti-IL-1β	BioLegend	Cat: 511710	Clone: H1b-98	100:1
anti-ST2	MD Bioproducts	Cat: 101002B	Clone: B4E6	200:1
anti-E-cadherin	Invitrogen	Cat: 13-1700	Clone: HECD-1	400:1
