# Supplementary material for: Cigarette smoke aggravates asthma by inducing memory-like type 3 innate lymphoid cells
Source: Nat Commun. 2022 Jul 4;13:3852. doi: 10.1038/s41467-022-31491-1 (PMC9253141; doi:10.1038/s41467-022-31491-1)
Supplement: Supplementary file 3 — Reporting Summary [file 41467_2022_31491_MOESM3_ESM.pdf]

nature portfolio

Corresponding author(s): Hye Young Kim and Jae-Woo Jung

Last updated by author(s): June 2, 2022

Reporting Summary

Nature Portfolio wishes to improve the reproducibility of the work that we publish. This form provides structure for consistency and transparency in reporting. For further information on Nature Portfolio policies, see our Editorial Policies and the Editorial Policy Checklist.

Statistics

For all statistical analyses, confirm that the following items are present in the figure legend, table legend, main text, or Methods section.

n/a	Confirmed	
☐	☒	The exact sample size (n) for each experimental group/condition, given as a discrete number and unit of measurement
☐	☒	A statement on whether measurements were taken from distinct samples or whether the same sample was measured repeatedly
☐	☒	The statistical test(s) used AND whether they are one- or two-sided<br>*Only common tests should be described solely by name; describe more complex techniques in the Methods section.*
☐	☒	A description of all covariates tested
☐	☒	A description of any assumptions or corrections, such as tests of normality and adjustment for multiple comparisons
☐	☒	A full description of the statistical parameters including central tendency (e.g. means) or other basic estimates (e.g. regression coefficient) AND variation (e.g. standard deviation) or associated estimates of uncertainty (e.g. confidence intervals)
☐	☒	For null hypothesis testing, the test statistic (e.g. F, t, r) with confidence intervals, effect sizes, degrees of freedom and P value noted<br>*Give P values as exact values whenever suitable.*
☒	☐	For Bayesian analysis, information on the choice of priors and Markov chain Monte Carlo settings
☒	☐	For hierarchical and complex designs, identification of the appropriate level for tests and full reporting of outcomes
☐	☒	Estimates of effect sizes (e.g. Cohen's d, Pearson's r), indicating how they were calculated

Our web collection on statistics for biologists contains articles on many of the points above.

Software and code

Policy information about availability of computer code

Data collection	No software was used to collect data
Data analysis	GraphPad Prism 7 was used for statistics. FlowJo V10 was used for analysis of FACS data. Image J was used for analysis of confocal data.

For manuscripts utilizing custom algorithms or software that are central to the research but not yet described in published literature, software must be made available to editors and reviewers. We strongly encourage code deposition in a community repository (e.g. GitHub). See the Nature Portfolio guidelines for submitting code & software for further information.

Data

Policy information about availability of data

All manuscripts must include a data availability statement. This statement should provide the following information, where applicable:

- Accession codes, unique identifiers, or web links for publicly available datasets
- A description of any restrictions on data availability
- For clinical datasets or third party data, please ensure that the statement adheres to our policy

Source data are provided with this paper. The source data underlying all reported averages in graphs underlying Figs. 1b-f, 2b, c, e, g, h, 3a–c, e-h, 4a-f, 5a–h, 6a–h and Supplementary Figs. 1-10 are provided as a Source Data file.

Human research participants

Policy information about studies involving human research participants and Sex and Gender in Research.

Reporting on sex and gender	Our study dose not containing sex and gender specific analysis
Population characteristics	We recruited 91 asthma patients (criteria: FEV1 changed over 12 % and 200 mL after bronchodilator response, and/or there was significant airway hyperresponsiveness to methacholine or mannitol provocation) and 24 healthy controls (without any respiratory diseases). All subjects were adults (age > 19) and Korean. 69 subjects were male, and 46 subjects were female.
Recruitment	Asthma patients were recruited from patients who visited Seoul National University Hospital and Chung-Ang University Hospital between December 2016 and June 2017, agreed to the study, and met some criteria: FEV1 changed over 12% and 200mL after bronchodilator response, significant airway hyperresponsiveness to methacholine or mannitol provocation, and without other diseases, such as cancer, severe medical conditions, or other pulmonary diseases. Patients on medications such as antibiotics, antifungal agents, antiviral drugs, probiotics, or any systemic steroid, were excluded because such medications might cause the change of immune responses.<br>Healthy donors who are without any respiratory diseases were recruited through a notice which was posted on the bulletin board of Seoul National University Hospital and Chung-Ang University Hospital during the same period of patients recruitment.
Ethics oversight	All subjects enrolled in this study provided written informed consent. The study protocol was approved by the Chung-Ang University Hospital Institutional Review Board (IRB number 1600-002-253) and the Seoul National University Hospital Institutional Review Board (IRB number 1608-163-788).

Note that full information on the approval of the study protocol must also be provided in the manuscript.

Field-specific reporting

Please select the one below that is the best fit for your research. If you are not sure, read the appropriate sections before making your selection.

☒ Life sciences ☐ Behavioural & social sciences ☐ Ecological, evolutionary & environmental sciences

For a reference copy of the document with all sections, see nature.com/documents/nr-reporting-summary-flat.pdf

Life sciences study design

All studies must disclose on these points even when the disclosure is negative.

Sample size	For patients cohort study, no sample-size calculation was performed because sample size was not chosen based on statistics and solely on availability of patient specimens. For in vitro experiments, an appropriate number of technical and biological replicates were generated under similar conditions yielding statistically significant results between groups.
Data exclusions	No data were excluded from the study.
Replication	In vitro experiments were performed multiple times with biological replicates as noted in the figure legends.
Randomization	Randomization was not applicable to the present cohort study. For all in vitro experiments of this study, randomization is not relevant. However, all experimental conditions were carefully controlled to ensure same experiment conditions were used for all groups.
Blinding	Blinding was performed during recruitment of subjects. All flow cytometry data were obtained before grouping the subjects. After finishing the recruitment of all the subjects, we divided the data according to their groups and performed statistical analysis.

Reporting for specific materials, systems and methods

We require information from authors about some types of materials, experimental systems and methods used in many studies. Here, indicate whether each material, system or method listed is relevant to your study. If you are not sure if a list item applies to your research, read the appropriate section before selecting a response.

Materials & experimental systems

n/a	Involved in the study
	☒ Antibodies
	☒ Eukaryotic cell lines
☒	Palaeontology and archaeology
☒	Animals and other organisms
☒	Clinical data
☒	Dual use research of concern

Methods

n/a	Involved in the study
☒	ChIP-seq
	☒ Flow cytometry
☒	MRI-based neuroimaging

Antibodies

Antibodies used

Percp/cy5.5-labeled anti-human CD45, BD Bioscience (Cat.: 564105, Clone: HI30, Lot: 9135866)
FITC-labeled anti-human CD3ε, BioLegend (Cat: 300406, Clone: UCHT1, Lot: B279208)
FITC-labeled anti-human CD11c, BioLegend (Cat: 301604, Clone: 3.9, Lot: B276828)
FITC-labeled anti-human CD11b, BioLegend (Cat: 301330, Clone: ICRF44, Lot: B272326)
FITC-labeled anti-human CD14, BioLegend (Cat: 325604, Clone: HCD14, Lot: B268830)
FITC-labeled anti-human CD19, BioLegend (Cat: 302206, Clone: HIB19, Lot: B274550)
FITC-labeled anti-human CD49b, BioLegend (Cat: 359306, Clone: P1E6-C5, Lot: B220976)
FITC-labeled anti-human FcεRIα, BioLegend (Cat: 334608, Clone: AER-37, Lot: B226717)
FITC-labeled anti-human CD68, BioLegend (Cat: 333806, Clone: Y1/82A, Lot: B252924)
BV421-labeled anti-human CD117, BioLegend (C-Kit, Cat: 313216, Clone: 104D2, Lot: B274614)
PE/Cy7-labeled anti-human CD127, BioLegend (IL-7R, Cat: 351320, Clone: A019D5, Lot: B276506)
PE-labeled anti-human CD206, BioLegend (Cat: 321106, Clone: 15-2, Lot: B191529)
APC-labeled anti-human HLA-DR, BioLegend (Cat: 307610, Clone: L243, Lot: B278650)
BV650-labeled anti-human CD4, BioLegend (Cat: 317436, Clone: OKT4, Lot: B284308)
BV510-labeled anti-human CD45RO, BioLegend (Cat: 304245, Clone: UCHL1, Lot: B235648)
APC-labeled anti-human CD45RA, BioLegend (Cat: 304150, Clone: HI100, Lot: B256627)
PE-labeled anti-human CD56, BioLegend (Cat: 318305, Clone: HCD56, Lot: B243218)
APC-labeled anti-human CD16, BioLegend (Cat: 302012, Clone: 3G8, Lot: B279345)
APC-labeled anti-human NKp44, BioLegend (Cat: 325110, Clone: P44-8, Lot: B254261)
PE/Cy7-labeled anti-human IFNγ, BioLegend (Cat: 502527, Clone: 4S.B3, Lot: B236362)
BV421-labeled anti-mouse/human IL-5, BioLegend (Cat: 504311, Clone: TRFK5, Lot: B325345)
APC-labeled anti-human IL-17A, BioLegend (Cat: 512333, Clone: BL168, Lot: B234864)
Pacific blue-labeled anti-human IL-1β, BioLegend (Cat: 511710, Clone: H1b-98, Lot: B316030)
Biotinylated anti-human ST2L, MD Bioproducts (Cat: 101002B, Clone: B4E6, Lot: S1606003-D)
E-cadherin monoclonal antibody, Invitrogen (Cat: 13-1700, Clone: HECD-1)

Validation

Commercial antibodies were validated by the manufacturer's as indicated on their websites.
anti-human CD45 (BD Bioscience, 564105)
https://www.bdbiosciences.com/en-tw/products/reagents/flow-cytometry-reagents/research-reagents/single-color-antibodies-ruo/percp-cy-5-5-mouse-anti-human-cd45.564106
anti-human CD3ε (BioLegend, 300406)
https://www.biolegend.com/en-us/productstab/fitc-anti-human-cd3-antibody-863
anti-human CD11c (BioLegend, 301604)
https://www.biolegend.com/en-us/products/fitc-anti-human-cd11c-antibody-562
anti-human CD11b (BioLegend, 301330)
https://www.biolegend.com/en-us/products/fitc-anti-human-cd11b-antibody-8299
anti-human CD14 (BioLegend, 325604)
https://www.biolegend.com/en-us/products/fitc-anti-human-cd14-antibody-3951
anti-human CD19 (BioLegend, 302206)
https://www.biolegend.com/en-us/products/fitc-anti-human-cd19-antibody-717
anti-human CD49b (BioLegend, 359306)
https://www.biolegend.com/en-us/products/fitc-anti-human-cd49b-antibody-8880
anti-human FcεRIα (BioLegend, 334608)
https://www.biolegend.com/en-us/products/fitc-anti-human-fcepsilonrialpha-antibody-5059
anti-human CD68 (BioLegend, 333806)
https://www.biolegend.com/en-us/products/fitc-anti-human-cd68-antibody-4844
anti-human CD117 (BioLegend, 313216)
https://www.biolegend.com/en-us/products/brilliant-violet-421-anti-human-cd117-c-kit-antibody-7348
anti-human CD127 (BioLegend, 351320)
https://www.biolegend.com/en-us/products/pe-cyanine7-anti-human-cd127-il-7ralpha-antibody-7216
anti-human CD206 (BioLegend, 321106)
https://www.biolegend.com/en-us/products/pe-anti-human-cd206-mmr-antibody-2994
anti-human HLA-DR (BioLegend, 307610)
https://www.biolegend.com/en-us/products/apc-anti-human-hla-dr-antibody-787
anti-human CD4 (BioLegend, 317436)
https://www.biolegend.com/en-us/products/brilliant-violet-650-anti-human-cd4-antibody-7786
anti-human CD45RO (BioLegend, 304245)
https://www.biolegend.com/en-us/products/brilliant-violet-510-anti-human-cd45ro-antibody-11922
anti-human CD45RA (BioLegend, 304150)

https://www.biolegend.com/en-us/products/apc-anti-human-cd45ra-antibody-684
anti-human CD56 (BioLegend, 318305)
https://www.biolegend.com/en-us/products/pe-anti-human-cd56-ncam-antibody-3796
anti-human CD16 (BioLegend, 302012)
https://www.biolegend.com/en-us/products/apc-anti-human-cd16-antibody-565
anti-human NKp44 (BioLegend, 325110)
https://www.biolegend.com/en-us/products/apc-anti-human-cd336-nkp44-antibody-3850
anti-human IFNγ (BioLegend, 502527)
https://www.biolegend.com/en-us/products/pe-cyanine7-anti-human-ifn-gamma-antibody-5939
anti-mouse/human IL-5 (BioLegend, 504311)
https://www.biolegend.com/en-us/products/brilliant-violet-421-anti-mouse-human-il-5-antibody-9392
anti-human IL-17A (BioLegend, 512333)
https://www.biolegend.com/en-us/products/apc-anti-human-il-17a-antibody-9987
anti-human IL-1β (BioLegend, 511710)
https://www.biolegend.com/en-us/products/pacific-blue-anti-human-il-1beta-antibody-5836
anti-human ST2L (MD Bioproducts, 101002B)
https://www.mdbioproducts.com/products/st2l-human-monoclonal-antibody-biotinylated?variant=39848198668477
E-cadherin monoclonal antibody (Invitrogen, 13-1700)
https://www.thermofisher.com/antibody/product/E-cadherin-Antibody-clone-HECD-1-Monoclonal/13-1700

Eukaryotic cell lines

Policy information about cell lines and Sex and Gender in Research

Cell line source(s)	A549 cell line was acquired from Korean Cell Line Bank (KCLB). RPMI2650 cell line was acquired from Korean Cell Line Bank (KCLB). BEAS-2B cell line was acquired from ATCC.<br>MLE12 cell line was acquired from ATCC.
Authentication	All cell lines were not authenticated.
Mycoplasma contamination	All cell lines were negative for mycoplasma.
Commonly misidentified lines<br>(See ICLAC register)	No commonly misidentified cell lines were used.

Flow Cytometry

Plots

Confirm that:

☒ The axis labels state the marker and fluorochrome used (e.g. CD4-FITC).

☒ The axis scales are clearly visible. Include numbers along axes only for bottom left plot of group (a 'group' is an analysis of identical markers).

☒ All plots are contour plots with outliers or pseudocolor plots.

☒ A numerical value for number of cells or percentage (with statistics) is provided.

Methodology

Sample preparation	To eliminate mucus from sputum samples, the same volume of 0.1% dithiothreitol (Sigma, MO, USA) was added, the tube was shaken for 20 minutes at 37°C, and the mixture was filtered through a 70 µm strainer. After centrifugation at 1400 rpm for 6 minutes, the cell pellet was resuspended with 100 µLFACS buffer (PBS + 2% fetal bovine serum) for staining. Peripheral blood (PB) mononuclear cells (PBMC) were isolated by using Ficoll-Paque PLUS density gradient media (GE Healthcare, IL, USA). Briefly, PB was centrifuged at 2000 rpm for 10 minutes at 4°C to separate the plasma from the cells. After removing the plasma, the remaining cell pellet was resuspended with PBS and loaded onto a Ficoll-Paque layer. After centrifuging at 1800 rpm for 30 minutes, the PBMC layer was collected and washed with PBS.
Instrument	Flow cytometry was performed using BD LSRFortessaTM and BD LSRFortessaTM X-20 (BD, NJ, USA).
Software	Data were analyzed by FlowJo (V10) software (BD, NJ, USA).
Cell population abundance	No post-sort fractions were collected.
Gating strategy	ILCs: CD45+Lineage-IL-7R+ lymphocytes, after which the ILC1s (ST-2-C-kit- cells), ILC2s (ST-2+ cells), and ILC3s (ST-2-C-kit+ cells) were identified; CD4+ T cells: CD45+Lineage+CD4+ lymphocytes; Dendritic cells (DC): CD45+CD11c+HLA-DR+ cells; natural killer (NK) cells: CD45+CD11c-HLA-DR-CD56+ cells; classical monocytes: CD45+CD11c-HLA-DR+CD56-CD14+CD16- cells; intermediate monocytes: CD45+CD11c-HLA-DR+CD56-CD14+CD16+ cells; non-classical monocytes: CD45+CD11c-HLA-DR+CD56-CD14-CD16+ cells; macrophages: CD45+CD68+ cells; among macrophages, CD11c+CD206- cells are M1 macrophages and CD11c-CD206+ cells are M2 macrophages.

☒ Tick this box to confirm that a figure exemplifying the gating strategy is provided in the Supplementary Information.
